# Harnessing Microbes for Sustainable Development: Food Fermentation as a Tool for Improving the Nutritional Quality of Alternative Protein Sources

**DOI:** 10.3390/nu12041020

**Published:** 2020-04-08

**Authors:** Anna Kårlund, Carlos Gómez-Gallego, Jenni Korhonen, Outi-Maaria Palo-oja, Hani El-Nezami, Marjukka Kolehmainen

**Affiliations:** 1 Institute of Public Health and Clinical Nutrition, University of Eastern Finland, P.O. Box 1627, FI-70211 Kuopio, Finland; anna.karlund@uef.fi (A.K.); carlos.gomezgallego@uef.fi (C.G.-G.); jenni.korhonen@uef.fi (J.K.); hani.el-nezami@uef.fi (H.E.-N.); 2 Business School, University of Eastern Finland, P.O. Box 1627, FI-70211 Kuopio, Finland; outi-maaria.palo-oja@uef.fi; 3 School of Biological Sciences, University of Hong Kong, Pok Fu Lam Road, Hong Kong, China

**Keywords:** food fermentation, protein digestibility, mineral availability, antinutritional factors, alternative protein sources

## Abstract

In order to support the multiple levels of sustainable development, the nutritional quality of plant-based protein sources needs to be improved by food technological means. Microbial fermentation is an ancient food technology, utilizing dynamic populations of microorganisms and possessing a high potential to modify chemical composition and cell structures of plants and thus to remove undesirable compounds and to increase bioavailability of nutrients. In addition, fermentation can be used to improve food safety. In this review, the effects of fermentation on the protein digestibility and micronutrient availability in plant-derived raw materials are surveyed. The main focus is on the most important legume, cereal, and pseudocereal species (*Cicer arietinum*, *Phaseolus vulgaris*, *Vicia faba*, *Lupinus angustifolius*, *Pisum sativum*, *Glycine max*; *Avena sativa*, *Secale cereale*, *Triticum aestivum*, *Triticum durum*, *Sorghum bicolor*; and *Chenopodium quinoa*, respectively) of the agrifood sector. Furthermore, the current knowledge regarding the in vivo health effects of fermented foods is examined, and the critical points of fermentation technology from the health and food safety point of view are discussed.

## 1. Introduction

Currently, the sustainability of protein production is one of the major challenges in food systems, as animal-based proteins may possess relatively high environmental and economic impacts [1]. To meet the multiple levels of sustainability, the protein sources need to represent good biodiversity; respect ecosystems; be nutritionally adequate, safe, and healthy; be culturally acceptable; be easy to access, reasonably priced, and economically fair [2]. Therefore, there is a need to replace some animal proteins with plant-based ones and to develop food technological solutions to improve the nutritional quality of plant-based proteins, also providing high levels of essential micronutrients.

In contrast to animal-based proteins, specific cell wall components of plant whole food matrix, such as dietary fibers and phytochemicals, travel incorporated with the plant-based proteins through the digestive system and may interfere with protein digestibility [3]. Antinutritional factors (ANF) are plant-derived components that usually are considered as nutrient-availability-hindering agents and, therefore, as a target of elimination in the food technological solutions [4]; however, recently, the discussion on the potential health-promoting bioactivities of ANFs has been increasingly initiated [5,6,7]. Furthermore, although the amino acid composition—regarded as the most pivotal shortage of plant proteins from the nutritional quality point of view—cannot be altered by food technological means, food processing can be utilized to modify plant cell structures and components and thus to improve the digestive enzyme accessibility of plant proteins [4].

Fermented foods and beverages might have been a part of the human diet since the appearance of the human race [8], and there are thousands of different fermented foods and beverages around the world, most of them traditionally produced and locally consumed [9]. Fermented foods are foods or beverages made through controlled microbial growth and enzymatic conversions of major and minor food components [10,11]. Lactic acid bacteria (LAB), such as species belonging to *Lactococcus* and *Lactobacillus* genera, are the most commonly used and applied microorganisms, yet others, including yeast and molds, are also widely applied [9,12,13]. LAB are dominant players in most fermentation processes, and they produce a number of antimicrobial agents including metabolic products such as short-chain organic acids, carbon dioxide, hydrogen peroxide, lactoperoxidase, diacetyl, and other inhibitory substances [10,14]. All these produced substances cumulatively affect the fermentation process in its different phases, acting as antagonistics towards unwanted spoilage and opportunistic pathogenic organisms, while at the same time denaturing the proteins [8].

The industrialization of food production over the past century has reduced the diversity of fermented foods, particularly in the developed countries [10]. However, recently, the development of functional foods has been a main innovation trend in contemporary food markets, often with a particular interest in fermented foods [15]. In addition, fermentation has been considered as a potential way to improve nutritional quality of foods typically consumed in developing countries facing malnutrition issues [16]. Considering the food market, yogurt and fermented dairy products may be the most popular among consumers, but fermented cereals, legumes, vegetables, and fruits have recently attracted consumers’ attention, regaining popularity [15]. One of the main benefits of fermented food is the ingestion of beneficial microbes that can contribute to intestinal microbiota populations [17,18] or can impact resident microbial communities via different mechanisms: through trophic interactions, a direct alteration in fitness, or an indirect alteration in fitness through altered production of host-derived molecules [19].

In the view of the above, the objective of this review is to examine the quality-improving potential of food fermentation, in order to support the development of sustainable, nutritionally well-balanced, and safe alternative protein sources. The main focus is on the most important legume, cereal, and pseudocereal species of the agrifood sector, representing the most plausible raw material candidates for traditional and novel fermented plant-based protein sources. The effects of the fermentation process on the antinutritional factors and on the availability of dietary protein and micronutrients are surveyed in this paper.

## 2. Fermentation Process

Food fermentation processes can be categorized by the primary metabolites and microorganisms involved: alcohol and carbon dioxide (yeast), acetic acid (*Acetobacter*), lactic acid (LAB), propionic acid (*Propionibacterium*), butyric acid (*Clostridium*), and ammonia and fatty acids (*Bacillus*, molds) [10]. 

The combination of different raw materials and the fermentation process are responsible for the existence of more than 3500 traditional fermented foods worldwide [20]. Although traditional fermentations are guided for few dominant taxa, strain differences and population dynamics during the process can be remarkably complex; and minor changes can result in significantly different food products with variations in quality and organoleptic properties. At the industrial level, this process must be tightly controlled, and microbial starters with temporal and spatial stability and resilience are needed to produce high-quality foods with low variability between batches [10]. The main microorganisms and enzymes for the fermentation process have been clearly identified, mainly belonging to the genera *Lactobacillus, Lactococcus, Enterococcus, Vibrio, Weissella, Pediococcus, Enterobacter, Salinivibrio, Acinetobacter, Macrococcus, Kluyvera*, and *Clostridium* [21]. Nowadays, with the application of metagenomic approaches, it is possible to identify a vast array of microorganisms that are difficult to culture or that have never been previously isolated in fermented food, and it is also possible to have a clear profile and dynamics of the fermentation process [22].

Food fermentation confers certain advantages [9,10]: (1) food preservation due to the changes in the pH and the presence of antimicrobial products such as organic acids, ethanol, and bacteriocins; (2) changes in taste and texture, enriching organoleptic properties; (3) specific benefits depending on the food matrix and type of fermentation such as increasing bioavailability of nutrients or removal of undesirable compounds, like toxic components and antinutrients.

Despite many positive effects of the fermentation process, potential microbiological problems and health hazards may be driven by poor sanitary and hygiene conditions. These include biogenic amines [23], pathogenic and toxigenic bacteria [24], as well as mycotoxins metabolized by certain molds, even in the case of well-performed fermentation, when the quality of raw materials is low. Adverse effects associated with the consumption of fermented foods may sometimes be underestimated and should be carefully considered during the production of fermented foods.

The fermentation process can affect macronutrient composition. For example, several LAB exert amylolytic activity during the fermentation process, contributing to starch hydrolysis, and may increase digestibility and energy density of the fermented food [25], while others can reduce the starch availability [26]. Moreover, several publications confirm the increase in protein digestibility and content of free amino acids after fermentation in different food matrices [27,28,29]. This effect on protein digestibility may be a general effect for most of the food fermented with LAB and has been reported in different fermented foods, such as sourdough, with sprouted flour and quinoa yogurt-like products after fermentation with *Lactobacillus rossiae* LB5, *Lactobacillus plantarum* 1A7, *Lactobacillus sanfranciscensis* DE9, *Lactobacillus rhamnosus* SP1, *Weissella confusa* DSM 20194, and *Lactobacillus plantarum* T6B10 [26,30]. It is important to consider that fermentation can increase protein digestibility; meanwhile, some bacterial strains can use and reduce the amount of some essential amino acids, reducing the nutritional value of these proteins [31]. Considering the production purposes, if the objective is a beneficial modulation of protein digestibility, it is important to carefully select starter cultures that increase protein digestibility maintaining or increasing the nutritional value, releasing and synthetizing essential amino acids and not consuming them.

Fermentation can also result in new compounds with nutritional and health impact such as lactate, group B vitamins, essential amino acids and derivatives, several bioactive compounds such as bioactive peptides, polysaccharides, more bioavailable isoflavones, γ-aminobutyric acid, and antioxidants compounds, among others [10]. In tempeh, mold-fermented soybean food, the contents of folic acid, niacin, riboflavin, nicotinamide, and pyridoxine are found to be increased by *Rhizopus oligosporus*, whereas vitamin B12 is synthesized by nonpathogenic strains of *Klebsiella pneumoniae* and *Citrobacter freundii* [28]. Contents of thiamine, riboflavin, and methionine in idli, a rice-legume based fermented food, are enhanced during fermentation [32]. Similarly, vitamins B complex and C, lysine and tryptophan, and iron contents have been found to increase during fermentation of pulque, an alcoholic drink made from cactus plant [28]. In general, riboflavin and niacin contents are increased in many *Bacillus*-fermented foods [28], and riboflavin and folic acid were found to be synthesized in kimchi by *Lc. mesenteroides* and *L. sakei* [33]. Yeasts such as *Saccharomyces cerevisiae*, *Candida tropicalis*, *Aureobasidium* sp., and *Pichia manschuria* isolated from fermented cereal foods can produce vitamin B12 depending on raw material and fermentation conditions [34].

## 3. The Benefits of Food Fermentations from the Sustainability Point of View

### 3.1. The Multiple Levels of Food Production Sustainability

For the goals of sustainable development to be fulfilled, it is postulated that the nutritional sufficiency and cultural acceptability of food should be ensured. Mainly, the sustainable food production from a climate change perspective is close to the following current nutritional recommendations in Western societies: choose more whole grain cereals, vegetables, fruits, and berries; choose more plant-based protein sources; and reduce the amount of meat or animal-based protein sources [2]. The rapid rise of lifestyle diseases; consumers’ overall health awareness; and the general enthusiasm of consumers, food service, and the food industry for natural, healthy and flavorful food also support the increasing interest towards fermented food [35,36]. In addition, scientific interest in gut microbiota [37] has accelerated this development. While traditional and well-known fermented foods such as yogurt, kefir, sauerkraut, kimchi, and miso have already found their way back to consumers’ dining tables, consumers are also looking for alternative intestinal health products [38]. Fermented fruit juices, protein- and cereal-based juices, as well as longer-fermentation artisan breads are some of the responses to this growing demand [38,39].

It has been estimated that currently there are about 3500 fermented products on the market [40], some also including bioactive components [41]. In 2016, the global market size of fermented foodstuff was valued at about 149.5 billion USD and it is expected to reach 205.5 billion USD by 2023 [42]. Europe is at the forefront of the market for fermented foods and ingredients: fermented foods are particularly popular in the United Kingdom and Germany, where the market is expected to continue to grow strongly in the coming years [43]. Unilever, McNeil Nutritionals, Danone, Valio, and Kyodo, for example, have launched a wide range of fermented functional dairy beverages [35]; however, the proliferation of vegetarian diets, lactose intolerance, allergy to milk proteins, high fat and high cholesterol content associated with fermented dairy products as well as consumers’ general desire to try a plant-based diet for health or environmental reasons have stimulated public interest in nondairy alternatives [29,44]. For example, fermented alternatives to artificially carbonated soft drinks have gained a foothold, especially in Europe [45]. Kombucha and other fermented beverages are particularly attractive to the millennials [43] who favor minimally processed products and are eager to explore new tastes and exotic flavors [39,46]. Similar development is seen in product markets. Currently, about 40% of European consumers opt for plant-based products and try to incorporate more plants into their diets or purchase vegetarian and vegan food only [47]. Together with the current trend to increase protein intake, shifting consumer behavior opens opportunities for fermented nondairy foods that are rich in protein content.

However, nutritional adequacy of the environmentally sustainable diets might put certain subpopulations, such as infants, children, and elderly in Western societies as well as some populations living in low-income countries, at risk of nutritional insufficiency if realized without proper planning [1]. Despite their recognized nutritional properties and health benefits, fermented foods are not specially included and recommended in dietary guidelines, except for yogurt and other fermented dairy products. An exception are the dietary guidelines in India which explicitly encourage the consumption of fermented foods [21]. However, it has recently been suggested that fermented foods should be considered as part of national dietary recommendations [48], with a special emphasis on those products that are part of the traditional diet of each population group. Beyond their nutritional role, the promotion of fermentation and the recovering of traditional fermented foods can have a societal role, promoting the development of disadvantaged communities and supporting the reduction of infections and diarrhea, for example [49,50,51,52,53]. Yogurts enriched with probiotic *Lactobacillus* or with *S. thermophilus* have been employed in some societal developing programs, but the production of cereal- and vegetable-based fermented foods has also been encouraged [49,51,52]. Indeed, in some populations, animal-based protein sources are vital in maintaining at least close to adequate nutritional status, for example due to shortage of fresh water to grow enough plant-based protein sources; however, fermentation processes can be utilized in attempts to increase the nutritional quality of local vegetarian foods.

### 3.2. Improving the Nutritional Quality of Sustainable Protein Sources by Reducing Antinutritional Factors

In the most recent scientific literature, the most widely studied ANFs interfering with protein and mineral availability are phytic acid, phenolic compounds, especially condensed tannins, and protease inhibitors.

In the physiological pH of the gastrointestinal tract, phytic acid—plants’ organic storage form of phosphorus—may form insoluble complexes with metal ions and thus reduce the bioavailability of important micronutrients, such as calcium (Ca), iron (Fe), magnesium (Mg), and zinc (Zn) [54]. To avoid mineral deficiencies in populations consuming diets high in phytic acid and/or susceptible to malnutrition, in general, novel and traditional food technological approaches are actively surveyed for phytic acid reduction [4,54]. Phytases are enzymes endogenously produced by plants but also by fermenting organisms [55,56,57]. Endogenous phytases are often activated by the reduction in pH, typically taking place during food fermentation, and usually contribute significantly to the phytic acid reduction in fermented plant matrices [57]. Phytase activity is also a desirable characteristic of starter culture bacteria, especially if the activity can be maintained in the physiological conditions of the human gastrointestinal tract [56]. However, there are major differences in the phytic-acid-reducing capacity of LAB; thus, in the future, genetic modifications may offer novel means to enhance the phytase activities of food-fermenting bacteria and to further develop food products with better nutritional quality and functionality [55].

Phenolic compounds are a large group of secondary plant metabolites widely appearing in food plants with variable qualitative and quantitative profiles. Foods providing high levels of phenolic compounds have been studied in terms of their various health benefits in in vitro, experimental, and clinical settings, as well as in regard to associations in epidemiological cohort studies. Although they are important components for food quality and stability [58], phenolic compounds have a potential to form complexes with proteinous molecules in the gastrointestinal conditions, thus interfering with protein bioavailability [59]. The interaction and binding between phenolic compounds and proteins, e.g., digestive enzymes and dietary proteins, depends not only on the structures of both the phenolics and proteins, but also on some environmental parameters, such as pH and temperature [58]. Condensed tannins, i.e., proanthocyanidins, are a subgroup of tannins with potential toxic effects on many organisms and relatively high tendency to precipitate protein molecules [60,61]. Microbial tannase enzymes have been evolved for biodegradation of tannins for energy production and toxicity reduction and are often expressed during fermentation processes of bacteria and fungi [60]. For example, *Lactobacillus plantarum* strains are commonly isolated from fermented plant materials, and many of them express tannase enzymes capable of tannin compound degradation [62].

The suggested biological roles of plant-derived protease inhibitors include pesticidal activities towards the digestive enzymes of foraging animals, as well as endogenous proteinase regulation and the storage of sulfur amino acids [63]. Cereals and legumes possess Kunitz and Bowman−Birk family trypsin and chymotrypsin inhibitors that are proteinous in nature and that bear reactive inhibiting sites in their amino acid chain [63,64]. Via these reactive sites, inhibitor molecules bind to the reactive sites of digestive enzymes, thus blocking their activity on food-derived proteins [64,65]. Protease inhibitors are often resistant to changes in pH and to the other physiological conditions prevailing in gastrointestinal tract [65]. Furthermore, their level may vary between plant cultivars [66]. Food technological means have been developed to inactivate the protease inhibitors, and the inactivation mechanisms include, for example, inhibitor aggregation and the cleavage of inhibitor’s peptide bonds [64], along with de novo synthesis of proteases [66]. Fermenting bacteria may also produce proteolytic enzymes capable of reducing protease inhibitors; here, the microbial source of the protease, process parameters, and the type and chemical composition of the food matrix have an impact on the efficacy of trypsin inhibitor removal by fermentation [67].

#### In Vitro Protein Digestibility as a Measure of Nutritional Quality

In vitro protein digestibility (IVPD) is a common tool in evaluating the nutritional quality of foods. In in vitro settings, the term digestibility often refers to the level of increase in the nitrogen not incorporated in protein structures, i.e., nonprotein nitrogen [27,68]. The higher the level of nonprotein nitrogen in the digested food matrix is, the better the digestibility is considered to be. Protein digestibility can also be determined by measuring soluble proteins [69] or protein hydrolysis, evaluated based on pH changes in digestive suspension [70] or on visual changes in gel electrophoresis [71].

Proteins need to be cut in smaller pieces before they can be absorbed and further used in different kinds of activities of the human body, and in vitro models can be used to make preliminary assessments on how the proteins (1) are cut in the real-life gastrointestinal system; (2) are absorbed; and (3) potentially execute some nutritional or bioactive operations [72]. The very basic static in vitro gastrointestinal models are affordable, fast to set up and use, and possess no ethical issues because no living creatures are utilized. More sophisticated dynamic models for simulations of human gastrointestinal system have also been developed [73]. However, static in vitro models can also be easily adjusted to simulate very different conditions; it is possible to mimic, for example, a healthy adult, an infant, an elderly person, or a disease condition and to study the fate of dietary components in the digestive tract [74].

## 4. The Effects of Fermentation on Nutritional Quality: Special Focus on Protein and Micronutrient Availability in Legume and Cereal Products

### 4.1. Legumes

Several members of the Fabaceae plant family, i.e., legumes, constitute a highly important group of food crops and are traditionally consumed in many countries and food cultures [75]. Legumes are good sources of dietary plant proteins and micronutrients, for example, group B vitamins, Ca, Mg, Fe, and Zn (Table S1) [76]. The awareness of health and global sustainability issues has increased the popularity of legumes among researchers, consumers, and food industry operators [75]. Furthermore, legume fermentation with either natural or starter-culture-based approach has drawn special attention, as the microbial metabolism and enzyme activities potentially help (1) to increase macro- and micronutrient bioavailability; (2) to decrease adverse gastrointestinal symptoms related with legume consumption, e.g., by reducing fermentable oligo-, di-, monosaccharides, and polyols (i.e., FODMAPs) promoting the symptoms of irritable bowel syndrome (IBS); and (3) to produce bioactive peptides with inhibitory activities towards metabolic-syndrome-associated enzymes. The relatively poor digestibility of legume proteins stems from ANFs, such as protease inhibitors which are widely expressed in the legume group [77]. Phytic acid and saponin and tannin compounds are also widely studied in legumes [75]. In addition, raffinose, along with other oligosaccharides (stachyose and verbascose), is one of the most abundant flatulence-causing FODMAPs in legumes [78]; thus, its content and reduction in legume-based raw materials has aroused high interest [75].

Fermented legumes can be consumed as traditional food products, such as tempeh [79], or produced to be used as an ingredient in other food products [77]. Lactobacilli, e.g., *L. acidofilus*, *L. brevis*, *L. delbruckerii*, *L. plantarum*, and *L. sakei*, are commonly utilized in legume fermentation, with *L. plantarum* being the most widely used. In addition, *Streptococcus thermophilus*, *Bifidobacterium*, and *Pediococcus* strains have been utilized. In tempeh-type products, *Rhizopus* molds are traditionally used as fermenting organisms. In scientific literature, the typical fermentation temperatures and times range between 30−42 °C and 11−96 h, respectively, depending on the fermenting organism and the raw material. Longer fermentation periods (e.g., 7 days) can be applied to produce special functionalities, for example, via bioactive peptide formation [80]. 

In this review, six of the economically most important *Fabaceae* family members on the EU level are surveyed: chickpea (*Cicer arietinum*), common bean (*Phaseolus vulgaris*), faba bean (*Vicia faba*), narrowleaf lupin (*Lupinus angustifolius*), pea (*Pisum sativum*), and soybean (*Glycine max*) [81]. All these grain legumes are in the top 10 of the most studied pulses in the world, reflecting their importance in agrifood sector [82]. For example, chickpea, faba bean, lupin, and pea cultivation in Europe has been recently more extensively supported by scientific community for the reduction of meat consumption and soy import, and it has been suggested that innovative product development in the plant protein sector could further enhance this positive trend [83]. In addition, common bean is widely cultivated and consumed in different climatic regions; due to the climatic change, its cultivation is postulated to increase in temperate regions, such as Denmark, the Netherlands, and the UK [84].

#### Fermentation Increases Nutrient Availability in Commonly Consumed Legumes

Yeast and LAB fermentation has been found to reduce the amount of phytic acid in faba bean [85], common bean [86], and soybean [87,88,89], for example. Furthermore, the reduction in phytic acid by LAB fermentation (e.g., strains *L. acidophilus* B4496, *L. bulgaricus* CFR2028, *L. casei* B1922, *L. plantarum* B4495, and *L. fermentum* B4655; 37 °C, 24 h) has been suggested to increase the Ca and Mg availability in soymilk [89]. Besides, LAB fermentation (24−96 h, 25 °C) of germinated soybean seeds with added saccharose has been found to increase the solubility of Ca, Mg, and Zn [90]; indeed, addition of sugars in the fermentation medium of soybeans might be crucial for some LAB strains and their mineral-solubility-enhancing metabolism, as the oligosaccharides of soybeans may not be well degraded and used as energy by these bacteria [55,90]. Although Fe availability might be reduced during soymilk fermentation [89], for soybean seeds it has been observed that germination prior to fermentation with lactobacilli may help to increase Fe solubility [90]. Furthermore, phytic acid/Fe molar ratio has been found to significantly decrease in fermented faba beans, which may imply an improvement in Fe bioavailability, although with some treatment parameters, the Fe content may also decrease [85]. In naturally fermented common bean flour and whole-bean samples (42 °C, 48 h), Fe, Mg, phosphorous, and potassium (K) have been found to sharply decrease, and Ca content was also slightly, although significantly, decreased [91]. In comparison to raw beans, Zn did not show any significant changes [91]. It was suggested that the reductions in mineral content may have resulted from the leaching into the fermentation water or and/or from microbial utilization [91].

When phytase activity in sourdoughs prepared from chickpea, pea, and kidney bean cultivars by *L. plantarum* V48 and *L. brevis* AM (24 h, 30 °C) were determined, it was observed that the enzyme activity was increased [92]. However, it was not further investigated whether this increase was caused by the activation of endogenous or bacterial enzymes.

In a study in which faba bean flours were fermented with *L. plantarum* VTT E-133328 strain (48 h, 30 °C) it was found that the treatment reduced the total amount of condensed tannins but that the total phenolics content was increased [77]. Similar trend was observed in a study surveying the effects of fermentation (24 h, 30 °C) of raw and gelatinized (i.e., cooked) flours produced from chickpea, pea, and black and white cultivars of common bean; in this study, the combination of *L. plantarum* MRS1 and *L. brevis MRS4* were used as the starter culture [75]. It was suggested that the coactivity of the enzymes from both the plant and the LAB caused the fractionation of condensed tannins to smaller phenolics [75,77]. Furthermore, sourdough fermentation of chickpea, pea, and kidney bean (*Phaseolus vulgaris*) (24 h, 30 °C) with *L. plantarum* V48 and *L. brevis* AM slightly but significantly reduced the amount of condensed tannins; at the same time, total phenolics were clearly increased, with some exceptions in the kidney bean group [92]. On the other hand, in a study using pea protein concentrate as a fermentation substrate for *L. plantarum* strain NRRL B4496, total tannin content, along with the total phenolic content, was found to increase; in this study, a fermentation period no longer than 11 h was applied [31]. The authors speculated this phenomenon to result from the liberation of tannin compounds from the lignocellulosic matrix during the fermentation process [31]. Thus, in addition to the selection of *L. plantarum* strains for legume fermentation, fermentation time may be a critical process parameter when aiming for tannin reduction. 

Interestingly, Shimelis et al. [93] noted that with natural fermentation, legume FODMAPs are efficiently reduced; this indicates some specific enzymatic activities of natural microflora, targeted for the breakdown of these oligosaccharides. However, it has been found that controlled fermentation of common bean with a mixed LAB culture (*L. acidofilus* LA-5, *Bifidobacterium* BB-12, *S. thermophilus* ABT-4; 4 days, 42 °C) does not reduce the content of raffinose and stachyose on a significant level [93]. Nevertheless, mixed-culture sourdough fermentation (*L. plantarum* V48, *L. brevis* AM; 24 h, 30 °C) of some chickpea and pea cultivars and several kidney bean cultivars has been shown to significantly decrease the level of raffinose [92], and a tempeh fermentation process with both *Rhizopus* mold and *L. plantarum* (31 h, 30 °C) also remarkably reduces the amount of raffinose and stachyose in common bean [79]. In addition, the content of verbascose in common bean was found to be reduced in combined tempeh fermentation, and a significant reduction in the contents of all studied oligosaccharides was also observed in the tempeh fermentations conducted only with *Rhizopus* [79]. In soybean, raffinose is somewhat stable at least for 48 h at 30 °C [94]. However, *Rhizopus* fermentation reduces the stachyose content of soybean, and verbascose is reduced to a minimum amount during a 48-h fermenting period at room temperature [94].

Fermentation of legumes in certain conditions and with specific bacterial strains may also lead to the formation of peptides with specific bioactivities [80,95]. For example, fermentation of pea seeds with *L. plantarum* for seven days at 22 °C was found to increase the potential for antihypertensive peptide formation during in vitro digestion of pea protein [80]. Same was observed for fermented *Phaseolus vulgaris* (cv. Eureka) seeds fermented at 30 °C for three days [95]. The processing parameters to produce bioactive peptides with certain desired activities should always be optimized specifically [95].

### 4.2. Cereals

Cereal foods, in general, are a fundamental component in human diets worldwide, providing the main energy source and a source of many essential nutrients [96]. Due to their high consumption, cereal foods constitute a crucial source of dietary protein, although the protein composition is not adequate when consumed alone without other sources of protein in the diet [97]. Whole grain cereals are, as part of a varied diet, convincingly and repeatedly associated with decreased risk of chronic disease morbidity and mortality [98,99], in addition to maintenance of balanced glucose and lipid metabolism and decreased low-grade inflammation [100,101,102,103]. However, proteins and other nutrients as well as bioactive compounds are located within cereal structures, such as in fiber fraction, and various technological approaches, such as fermentation, may be used to increase the bioavailability of these compounds and the nutritional value of the cereal foods. Sourdough breads, for example, are also widely consumed due to their well-known beneficial effects on the human glucose metabolism and health [96,104,105]. In addition, numerous types of fermented cereal beverages are widely prepared and consumed, especially in Africa, Asia, and Southern America [106]. 

In addition to cereals, pseudocereals have gained great interest, as the scientific data on their nutritional quality have accumulated [107]. In comparison to traditional cereals, quinoa (*Chenopodium quinoa*), for example, has a high protein content and balanced amino acid composition [53]; thus, although of Andean origin, quinoa has evoked high global interest as a novel protein source for sustainable food production [55,56]. Quinoa’s protein composition is surprisingly good, including most of the essential amino acids at the nutritionally valuable level [108]. Lysine, an essential amino acid commonly low in cereals, is included in quinoa in double the amount compared to wheat, for example. Thus, pseudocereals are also a promising source of energy and protein from a sustainability point of view.

In this review, traditional cereal crops: oats (*Avena sativa*), rye (*Secale cereale*), and wheat (*Triticum aestivum*, *Triticum durum*) have been considered for their importance in the European Union cereal crop sector production for human use [109]. In addition, the scientific literature surveying the fermentation of another Poaceae family member, sorghum (*Sorghum bicolor*), is reviewed. At the European level, sorghum is an emerging food crop with a high level of proteins and a desirable carbohydrate profile [109,110]. Therefore, sorghum is an important plant-based protein source contributing to food security in its original cultivation areas in Africa [111] and yet possesses high potential in supporting the public health of Western societies [110]. Although Peru and Bolivia are the largest producers of quinoa (*Chenopodium quinoa*) [112], quinoa is available for consumers, and can also be grown, almost all over the world [113]. For its high agroecological adaptability, quinoa has been acclimated in Europe [114], even as deep in the northern periphery as Finland [115]. Thus, in addition to cereals, studies regarding quinoa fermentations have been considered in this review.

#### 4.2.1. Applying Fermentation to Boost the Nutritional Quality of Cereal and Quinoa Products

In addition to flours, bran, flakes, and more refined oat fractions, oats are consumed as fermented beverages and yogurt-type products [116]. The protein content is relatively high in oats (Table S2), and the amino acid composition is well-balanced [117]. Due to high lysine level, oats have a good protein-digestibility-corrected amino acid score [16]. Fermentation with *L. plantarum* LP09 has been found to increase the amount of lysine and alanine in an oat beverage in comparison to nonfermented control product [118]. However, the scientific literature on the effects of cereal food fermentation on the oat protein digestibility and amino acid availability is rather scarce. In one study, the protein digestibility of oat grain was improved by fermentation with oyster mushroom *Pleurotus ostreatus* CS155 strain (2 weeks at room temperature), along with an increase in soluble nitrogen and a reduction in tannin content [119]. In addition, tempeh-type oat fermentation with filamentous fungi (*Aspergillus oryzae* var. *effuses* 3.2825, *A. oryzae* 3.5232 or *Rhizopus oryzae* 3.2751), for example, reduces phytic acid content within 72 h, which may enhance nutrient availability and protein solubility [120]. Furthermore, oat bran fermentation with baker’s yeast for 2−6 h at 30 °C has been found to reduce the phytic acid content, with 6-h incubation being the most effective [121]. 

Fungal fermentation with *Rhizopus oligosporus* ATCC 64063 has been found to preserve the mineral content (Fe, Zn) relatively well during oat beverage manufacturing and to reduce phytic acid content [122]. Yet, phytic acid content was speculated to stay on a level that may potentially hamper mineral absorption, and further processing was suggested to reduce the phytic acid content [122]. Addition of citric acid, phytase treatments, and Fe supplementation have been used to improve Fe availability in nonfermented oat beverages [123].

The literature concentrating on the colon fermentation of oats instead of food fermentation is more extensive. The fermentation of the in vitro digestion products of different oat cultivars with human fecal microflora has shown that colonic fermentation of oat flours produces high amounts of short-chain fatty acids (acetate, butyrate, and propionate) which could be beneficial for gut health [124]. Valerate and isovalerate, produced in small amounts in colonic oat fermentation, are short-chain fatty acids derived from proteins [124]; their physiological effects on the colonic cells of the host are not as well established [125]. Depending on the processing of different oat fractions, the growth and metabolism of variable types of probiotic bacteria isolated from human colon is enhanced [126]. Thus, for example, indigestible debranned oat fractions high in fiber can be considered as prebiotics promoting the growth and beneficial metabolism of different lactobacilli [126].

Rye is a major source of dietary fiber in Northern and Eastern European countries, and whole grain rye especially contains a reasonable amount of dietary protein [104]. In addition, rye contains high levels of micronutrients, such as group B vitamins, K, Mg, Fe, Ca, and Zn (Table S2), as well as various types of phytochemicals, for instance, lignans and phenolic acids [127,128]. 

Sourdough fermentation with *Candida milleri* C-96250, *L. brevis* E-95612, and *L. plantarum* E-78076 (20 h, 32 °C) has been found to increase the amount of amino acids, their derivatives, and small peptides in rye sourdoughs and corresponding breads, indicating efficient protein hydrolysis during the fermentation process [104]. In comparison to wheat bread, soluble proteins are at a higher level in sourdough rye breads, and the protein hydrolysis rate in in vitro conditions mimicking human gastric digestion is slower [96]. It has been shown that both the source and the degree of hydrolysis affect the absorption rate of dietary proteins [129], and this may lead to variable postprandial amino acid responses and bioactivities in vivo [96,104]. Sourdough rye bread has been found to contain, for example, branched-chain amino acids (potentially having beneficial effects on insulin metabolism) and bioactive peptides with possible antihypertensive and antioxidant effects [104]. 

However, rye also has a rather high content of phytic acid, tannins, and trypsin inhibitors, potentially reducing protein and mineral bioavailability [127,130]. In a study surveying the effects of fermentation process on rye bread phytic acid content and phytase activity, phytic acid was almost completely degraded in the sourdoughs, due to the efficient and prolonged phytase activity during the fermentation period of 10−12 h [128]. Phytic acid contents measured in the baked breads were also still significantly lowered in comparison to nonfermented dough stages, although some increase was observed in breads fortified with pressed rye grains [128]. Furthermore, it has been speculated that fermentation with LAB may help to reduce ANFs in rye porridges, for example [130]. 

Due to its exceedingly high global consumption, wheat is one of the most important nutrient sources in the world; furthermore, wheat is also a major source of dietary protein (Table S2) [131]. However, traditional processing of wheat grains tends to remove micronutrients, such as Fe and Zn [132], and regarding the protein content, modern breeding strategies in combination with climate change may reduce the nutritional quality of wheat [133]. In addition, wheat, like other plant-based raw materials, contains ANFs potentially hindering nutrient availability [16]. Thus, food technological solutions are developed to overcome these problems in maintaining or increasing the yield of essential nutritional components of wheat; among these, fermentation is one of the most promising approaches [16].

For example, wheat bran is a valuable by-product of wheat milling and potentially contains high levels of protein and micronutrients; however, wheat bran may contain high amounts of ANFs, especially phytic acid, which might be reduced by fermentation [134]. Experiments utilizing *L. brevis* E-95612 and *Candida humilis* E-96250 for the fermentation (24 h, 30 °C) of wheat bran have shown that IVPD is increased in comparison to untreated bran [69]. Wheat bran fermentation was also observed to increase the amount of free amino acids and total phenolic compounds and, furthermore, to increase phytase activity; when correlation analysis was conducted, the increase in IVPD was found to be linked especially with the increase in free amino acids and total phenolics, but also with the increase in phytase activity [69].

In addition to enhanced IVPD, the release of small peptides and free amino acids from cereal matrices during fermentation processes could increase the availability of proteins in the small intestine and thus contribute to the nutritional quality of the product as a protein source [135]. For example, it has been observed that proteins and peptides lower in molecular weight are present in the whole meal wheat breads that have undergone a sourdough fermentation process (19.5 h, 30 °C), in comparison to nonfermented whole meal wheat bread and white wheat bread [105]. Due to the pH drop occurring during yeast and lactic acid fermentation, the amount of soluble protein in wheat matrices also tends to increase [105,135]. However, fermentation of wheat slurry with *L. plantarum* CRL 778 (24 h, 30 °C) did not cause any significant changes in the amounts of free amino acids [135]. Furthermore, the protein digestion efficiency in in vitro gastric conditions did not differ between the bread types in the experiment surveying the effects of sourdough fermentation on wheat [105]. 

Interestingly, trypsin inhibitory activity (TIA) was not detected in the aqueous extracts of whole wheat flour or in corresponding extracts of whole wheat sourdough and bread, fermented with yeast (*Saccharomyces cerevisiae*; 30−31°C, 30 min) [136]. However, similar chymotrypsin inhibitory activities (CIA) were observed in whole wheat flour and sourdough, and the activity was sharply increased after baking the sourdough into bread; the authors suggested that novel protein fragments with CIA were formed during the sourdough fermentation [136]. Furthermore, TIA was detected in wheat sourdough bread digested with gastric and intestinal digestive enzymes in vitro, probably due to the release of these inhibitors from the bread matrix as a result of the enzymatic events [136].

Sorghum, although a widely used and nutritious stable food (Table S2), contains ANFs, for example, trypsin inhibitors, phytic acid, and tannin compounds; thus, reduction of these compounds by food processing is of high interest for the increased protein and mineral availability [70]. Natural fermentation (24 h, 37 °C) has shown potential in reducing, for example, the tannin content in sorghum [70]. With processing periods of 72−96 h, phytic acid in different sorghum varieties can also be effectively hydrolyzed by natural fermentation, and consequently, the solubility of Fe, Ca, Mg, and manganese is improved [137]. In addition, it has been found that fermentation (8 h, 37 °C) with a mixed LAB starter culture derived from milk and cultivated on soy whey medium, provides an efficient tool to reduce tannin compounds in sorghum grain (cv. Dadar), especially if the fermentation is followed by steaming and flaking [138]. Both natural (24 h, 37 °C) and starter culture-based (8 h, 37 °C) fermentation also helps to reduce TIA in sorghum [70,138], and a postfermentation steaming step further enhances this effect when the starter culture is used [138]. It should be considered that some cultivar-dependent differences in the level of ANF reduction may occur; for example, sorghum cultivar Shehla has shown lower level of reduction (15% reduction) in tannin content during natural fermentation in comparison to Hamra and Baidha (31% and 35% reduction, respectively) [70].

Natural fermentation of sorghum has been observed to increase IVPD progressively throughout a 24−28 h fermentation period at 37 °C, probably due to the reduction in ANFs [68,70]; after 28 h of fermentation, however, IVPD has been observed to level off [68]. Although sorghum is, in general, relatively poor in essential amino acids, fermentation may increase the levels of both essential and nonessential amino acids in sorghum-based medium and may improve the sorghum protein efficiency ratio and the biological value of sorghum grain proteins [138]. Nevertheless, in one study surveying the impact of both natural fermentation and starter-culture fermentation, it was observed that free amino acids were mostly reduced during the 24-h preparation process (at 30 °C) of sorghum-based fermented food togwa [139]; the starter cultures (*L. brevis*, *L. cellobiosus*, *L. fermentum*, *L. plantarum*, or *Pediococcus pentosaceus;* or combined culture of yeast *Issatchenkia orientalis* and *L. plantarum* or *P. pentosaceus*) consisted of LAB and yeast extracted from a similar product [139]. It is possible that the microbes present in these togwa-specific microbial cultures utilized the amino acids in their own metabolic activities [139]. However, the possibility of bioactive peptides to occur was speculated during togwa fermentation, as proteolytic activity was found to be high in the products, and high-molecular-weight proteins were effectively hydrolyzed [139]. 

Although fermentation may deteriorate the qualitative and quantitative profile of amino acids in togwa, fortification of nonfermented togwa gruel with malted sorghum was found to increase the level of free amino acids [139]; thus, further reductions in amino acids can potentially be prevented by adding malted sorghum to the gruel during preparation of fermented sorghum products. The effects of malting and fermentation have also been investigated in respect to vitamin B concentrations in sorghum slurries [140]. Natural fermentation (one week at room temperature) of malted red and white sorghum (cv. Seredo and -KARI Mtama 1, respectively) was effective in increasing the amount of group B vitamins in the slurry matrix: although malting alone had only little or no impact on the slurry vitamin contents, fermentation significantly increased the levels of folic acid, niacin, thiamin, pyridoxine (vitamin B6), and riboflavin [140].

Because fermentation is a traditional way to process cereals for improvements in nutritional and sensory quality, it is also more and more frequently applied to process pseudocereal quinoa [30,141,142,143,144,145]. Fermentations with *L. plantarum* strains have been found to increase Fe, Zn, and Ca solubility and to reduce phytic acid. For example, in a study surveying the effect of fermentation (16−18 h, 30 °C) with commercial *L. plantarum* starter, it was observed that the amount of soluble Fe in quinoa suspension was significantly increased in comparison to nonfermented suspension, while the content of phytic acid was significantly decreased [145]. Furthermore, fermentation (4, 10, or 48 h, 30 °C) of milled quinoa seeds with *L. plantarum* 299v has shown good efficiency in reducing phytic acid and a moderate capability to improve estimated Zn, Fe, and Ca availability [144]. These improvements were apparent already after 4 h [144]. Depending on the bacterial strains present in mixed LAB cultures (*Leuconostoc mesenteroides* subsp. *mesenteroides* CRL 2131; *L. plantarum* CRL 1964 and CRL 2107; *L. rhamnosus* CRL 1963, CRL 1984, and CRL 1983), the riboflavin and folate contents in fermented quinoa pasta doughs has been found to increase in variable levels; besides, phytic acid content was effectively decreased [142]. Quinoa sourdough, prepared using *L. plantarum* T6B10 and *L. rossiae* T0A16 (16 h, 30 °C) for bread making, has been found to have higher levels of total free amino acids and total phenolics in comparison to noninoculated quinoa dough, and phytase activity and IVPD were also increased; instead, condensed tannins were found to be decreased [146]. Fermentation of quinoa flour suspensions with *L. rhamnosus* SP1 or *L. plantarum* T6B10 (20 h, 30 °C) to produce yogurt-type products also resulted in an increase in total free amino acids and total phenolic compounds [30]. Furthermore, IVPD of the products, as well as the calculated essential amino acid indexes, protein scores, protein efficiency ratios, and biological values and nutritional indexes were markedly increased [30]. The observed improvements in nutritional values were even more pronounced after a 20-day storage period [30]. Fermentation with *Weissella confusa* DSM 20194 and added sucrose, too, increased IVPD, but on a lower level [30].

Fermentation of quinoa has often been combined with other food processing technologies, such as germination [145], milling [143], or roasting [144]. Typically, these pre- or post-treatments further decrease phytic acid and consequently increase mineral solubility; however, for instance, dry roasting after fermentation seems to be more efficient in aiding the phytic acid reduction and mineral availability, in comparison to dry roasting performed in advance [144]. It has been suggested that the pre- and post-treatments may help to either activate or inactivate endogenous phytases [144,145]. 

#### 4.2.2. Fortification of Fermented Cereal Food Products with Pseudocereal and Legume-Based Ingredients

Fermented legume and pseudocereal-based ingredients are sometimes used in an attempt to increase the nutritional value of cereal products, such as white wheat bread. In addition, side streams or by-products of other sectors of agriculture or food industry might be utilized in this respect. This is an important approach as, in many developing regions of the world, there is a need for affordable and nutrient-dense ingredients to improve the quality of traditional, plant-based dishes with inadequate or imbalanced micro- and macronutrient contents [27].

For instance, sorghum and faba bean, both readily available in many developing countries of Africa and Asia, can be combined as fermented ingredients to increase the potential protein availability of the end-product [27]. Indeed, IVPD in the combination of sorghum and faba bean flours was increased when natural fermentation (24 h, 37 °C) was applied on both types of flour; however, the authors noted that there might be some differing effects of fermentation on IVPD, depending on the sorghum and faba bean cultivars used [27]. Furthermore, natural co-fermentation (24, 48, and 72 h, at room temperature) of blended sorghum and soybean flours has been found to increase the production of B group vitamins thiamin, niacin, and riboflavin in baked cookies when compared to cookies prepared with nonfermented sorghum–soybean cookies [147]. The increasing effect was more pronounced in products with higher level of soybean (20%−25%) and that had been fermented for longer periods of time [147]. Instead, the contents of tannins, phytic acid, and protease inhibitors were observed to be decreased [147]. Tannins and phytic acid were found at their highest level in cookies prepared from 100% nonfermented sorghum flour; their levels decreased as the fermentation period and the portion of soybean increased [147]. Protease inhibitors, both trypsin inhibitors and total protease inhibitors, on the other hand, were increased along with the soybean portion, although the longer fermentation periods resulted in lower concentrations [147].

Quinoa sourdoughs have been used to replace wheat in white bread [146] and semolina pasta [148] and as a component in fermented wheat bread [114]. The conclusions in these experiments have been that quinoa sourdoughs have positive effects on the nutritional values of wheat products. For example, the concentration of total free amino acids, including lysine, in white bread and semolina pasta has been found to increase when quinoa sourdough (*L. plantarum* T6B10, *L. rossiae* T0A16; 16 h, 30 °C) has been added at 20% concentration [146,148]. Instead, the IVPD in white bread and pasta with fermented quinoa sourdough has been found to slightly decrease in comparison to the all-wheat products [146,148]. However, when the protein scores, essential amino acid indexes, protein efficiency ratios, biological values, and nutritional indexes of the products were calculated, the fermented quinoa-containing products were scored significantly higher; the amount of total phenolic compounds were also higher in comparison to the wheat bread and pasta doughs without fermented quinoa added [146,148]. Substitution of wheat with quinoa (25% or 50%) in sourdough bread (*S. cerevisiae*; 24 h, 4 °C, proofing at 28 °C) increases the mineral content; however, inclusion of whole quinoa flour resulted in an increase in phytic acid concentration [114]. It was shown that bifidobacterial phytases expressed in *Escherichia coli* reduce phytic acids in quinoa-containing breads when exogenously added, and this strategy was suggested for the future product developments [114].

Fermented faba bean, mixed and fermented with rapeseed and sunflower meals and/or with wheat bran, has been studied for its nitrogen and protein solubility [149]. The mixtures were supplemented with potato pulp containing a commercial LAB starter and incubated for 3−5 days at 35 °C in vacuum bags [149]. The researchers found that in the fermentation mixtures including the highest levels of wheat bran, the nitrogen and protein solubility was most effectively increased over time; it was suggested that the enzymes present in the bran were most susceptible to the impact of fermentation [149].

## 5. From In Vitro to In Vivo

Because of the complex roles, fates, and interactions of dietary and endogenous proteins in human physiology, the bioavailability of proteins in a meal presents a true and intriguing challenge to nutrition scientists. While the metabolism of carbohydrates and lipids can be measured with well-established methodology surveying human glycemic responses and adipose tissue metabolism [150,151,152], the utilization of proteins in human physiological phenomena cannot be followed in such a straightforward way.

To study the in vivo bioavailability of animal proteins, practices to intrinsically label protein amino acids with stable carbon-13 isotope have been developed, and these approaches are utilized to study the fate of milk, egg, and meat proteins in human metabolism [153,154,155,156,157]. In addition, some studies have employed nitrogen-15 labeling [158], or dual tracer [159] and indicator amino acid oxidation [160] methods to study the metabolic availability of plant proteins in humans. However, although evidence on the protein-digestibility-improving effects of food fermentations is accumulating, there are no meal studies surveying this phenomenon in real physiological conditions of the human gastrointestinal system.

Dynamic in vitro gastrointestinal models, such as TIM and SHIME, have been evaluated for their capability to mimic human in vivo conditions and are suggested to be rather valuable tools in this respect [73]. Static in vitro gastrointestinal models usually used for the IVPD evaluations are vague and thus inadequate in complexity for drawing conclusions regarding true in vivo effects [73], even though within one experiment, they may provide useful means to compare the effects of different food processing methods on protein digestibility, for example. Furthermore, harmonized static in vitro models have been developed, such as INFOGEST, aiming for better comparability of results gained in different laboratories [161]. In respect to fermented foods, in vitro approaches to study the survival of the starter culture microbes in the digestive environment can be useful; however, the dynamic pH and gastric emptying as well as the composition of human bile salts are hard to simulate here [162]. These challenges may lead to underestimation of microbial resistance to gastrointestinal digestion in vivo [162]. Nevertheless, some in vivo research on the nutrient availability in fermented foods has been conducted. 

Fermentation of oat gruel with *L. plantarum* 299v has shown positive effects on nonheme iron absorption in human subjects; as the phytic acid content, for example, stayed rather stable throughout the gruel processing experiment, the Fe absorption-improving effect was speculated to be related to the presence of the LAB [163]. In an animal study on male Wistar rats, natural sourdough fermentation of wheat was shown to be effective in improving the apparent absorption of Mg and copper, in comparison to nonfermented wheat flour; both yeast (*Saccharomyces cerevisiae*) and natural sourdough fermentation significantly increased the apparent absorption of Fe and Zn [164]. Interestingly, there was no effect on Ca absorption [164]. The improvement in apparent mineral absorption by yeast and sourdough fermentations was suggested to result from the reduction of phytic acid [164]. Furthermore, in a study focusing on the development of gluten-free pasta with high nutritional value, quinoa pasta prepared from a sourdough fermented with mixed LAB culture of *L. plantarum* CRL 2017 and *L. plantarum* CRL 1964 was able to improve the nutritional status in a mice model fed with a vitamin-deficient diet, in respect to folates and riboflavin [142]. In addition, the reduction in phytic acid implied a beneficial effect of quinoa fermentation process on in vivo mineral bioavailability [142]. It was suggested that the selected LAB strains were effective in producing folate, riboflavin, and phytases in the quinoa-based medium [142].

Because of their long history and high popularity in Asian countries, fermented soy products have been studied for their mineral and vitamin status-enhancing properties in vivo. For example, fermentation of soybean meal with *Aspergillus usamii* mold for 48 + 12 h has been found to increase the femur Zn content in male Wistar rats, in comparison to nonfermented soybean meal, indicating improved Zn bioavailability [165]. Again, the improvement of Zn solubility in the small intestine in vivo was associated with the reduction in phytic acid during fermentation [165]. In a similar study surveying the impact of *A. usamii* fermentation on the Zn and Fe bioavailability of soybean flour, Zn content in rat femur and plasma were found to increase in a dose-dependent manner when nonfermented soy flour and a combination of fermented and nonfermented soy flours were used as references [88]. Fe bioavailability measured from femur and plasma was also increased, and the level of increase was similar in both fermented and combination soy flour interventions [88]. Thus, for Fe bioavailability, it was discussed whether the complex formation tendency of phytic acid towards Fe was lower in comparison to Zn [88]. Overall, vitamin K_2_, i.e., menaquinone-7 (MK-7), produced by microbiota present in many dairy and fermented food products, is known to be high in bioavailability [166]. For instance, the fermented soy product natto is naturally high in MK-7, established to support Ca metabolism and osteoporosis prevention [167]. Indeed, when prolonged natto intake was studied in ovariectomized female Wistar rats, the Ca content of the diaphyseal femur was measured; the effect was highlighted when additional MK-7 was provided [167]. 

Although narrow-leaf lupin is now considered as a suitable raw material for human consumption, its traditional usage is in the animal feed sector as an alternative for soy protein [168]. Thus, the ileal digestibility of fermented narrow-leaf lupin cultivar Neptun has been studied in pigs [169]. Fermentation with baker’s yeast (*S. cerevisiae*) and a mixed culture of *Enterococcus faecium*, *L. plantarum*, *L. buchneri*, and *L. casei* was found to increase the apparent ileal digestibility of lupin seeds to a level similar to soybean meal, and the apparent ileal digestibility of amino acids arginine, isoleucine, and cysteine was increased to a significantly higher level in comparison to soybean meal [169].

In general, more refined protein fractions, such as isolates and extracts, of plant-based foods could be used as enrichments to increase the amount, quality, and/or sustainability of proteins in other foods and beverages [170,171,172]. Furthermore, plant protein extracts could potentially be further processed to added-value products or pharmaceuticals [171,172], and fermentation processes may serve as instruments to increase tailored health-supporting bioactivities. As an example, a wheat germ protein extract fermented with baker’s yeast has been found effective in reducing tumors related with non-Hodgkin lymphoma in an immunodeficient mouse model, probably via a natural killer cell regulated antitumor mechanism [173].

## 6. The Prospects of Research and Process Development in the Field of Food Fermentation

### 6.1. Process Optimization for Nutritionally Desirable Outcomes

**Figure 1:**
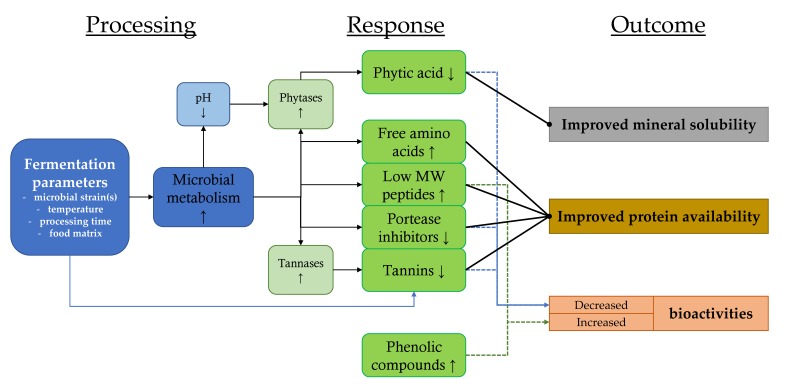
An overview on the effects of the fermentation process parameters and microbial metabolic events on the nutritional quality factors of plant food matrices. Although the overall effect of food fermentation on mineral solubility and protein availability is often positive, and especially protein-derived residues and phenolic compounds may contribute to desirable bioactivities (green dashed lines), the reduction in phytic acid, protease inhibitors, and tannin compounds may lead to a decrease in activities potentially beneficial for human health (blue dashed lines). MW, molecular weight.

Based on the reviewed literature, the effects of fermentation on legume protein digestibility, for example, are largely dependent both on the fermentation parameters and on the type of plant material [78]. However, in general, IVPD tends to increase as a result of fermentation, although the level of improvement varies greatly. Fermentation may also increase the amount of free amino acids in legume-based products, depending on the legume species and cultivar [92]. Usually, fermentation can be used to reduce the phytic acid content in the raw material. Meanwhile, effects on different classes of tannin compounds have been found to be variable, although fermentation usually lowers the amount of condensed tannins. In addition, inhibitory activity towards trypsin is also often, although not always, reduced [31,75,77,94,174]. Thus, as a result of overall reductions in phytic acid and/or tannin compounds and possibly other ANFs, protein digestibility and mineral solubility are enhanced. An overview of the effects of the fermentation process on the ANFs and on the nutrient availabilities is presented in Figure 1.

Sourdough fermentation helps to increase the level of free amino acids, including essential amino acids in cereal-based products; thus, the prospects of protein availability in the intestinal system could be enhanced (Figure 1). However, the endogenous and microbe-derived proteolytic and peptidolytic activities in sourdoughs resulting in the formation of smaller peptides and free amino acids in the end-products are often considered as quality-improving traits, usually from the sensory and technological points of view [175]. Most publications surveying fermented cereals from the well-being point of view concentrate on the role of major prebiotics and probiotics in the development of chronic diseases, or on the decreasing effect of sourdough fermentation in the symptomatology of IBS and different levels of gluten intolerance, although, due to their high consumption, cereal foods constitute a crucial source of dietary protein.

There is an increasing consumer demand for healthy snack products, optimally suitable for different special diets [141], and the efforts to develop functional, pre- and/or probiotic fermented cereal beverages for industrial production using known starter cultures have been intense in recent decades. However, for now, the research and development in the field of fermented cereal drinks and beverages concentrates on process optimization, on short-chain fatty acid production in the colon, and on the constancy of basic nutritional values and sensory properties. Fermented cereal beverages, in general, have been suggested to support the mitigation of the symptoms of IBS, diarrhea, constipation, and flatulence, for example [176]; as observed in maize, LAB fermentation may help to reduce, for example, the content of flatulence-causing oligosaccharides in other cereal products as well [130]. The first step in the successful development of functional cereal beverages is to determine the appropriate microbial strains to produce homogenous and functional products with acceptable sensory properties [106,177,178].

### 6.2. Critical Points

Although, on average, the capacity of food fermentation as a tool for improving the protein and mineral availability seems almost tremendous, it must be remembered that plant cultivars differ in their responses to fermentation, and for some raw materials, different processing parameters may work better for certain purposes than for others. In addition, one of the most controversial aspects of food fermentation is the reduction of the so-called antinutritional factors present in plant-based materials (Figure 1). Phenolic compounds especially, including tannins, are widely considered as health-promoting agents, and their reduction might be seen as a drawback. In addition, protease inhibitors are important regulators of many hormonal and metabolic events [7], and phytic acid is a natural antioxidant with suggested beneficial health effects [5]. Thus, it can be pondered whether the increase or decrease of these potentially bioactive compounds should be the goal of food technological solutions.

Minimally processed food without chemical preservatives labeled with “natural” or “clean-label” has gained consumers’ attraction in recent years in Western countries. In order to fulfil the high consumer demands, fermentation could be one of the most prominent methods for not only increasing the bioavailability of nutrients, but also increasing the shelf-life and minimizing the food waste. In cereal products, one of the main reasons for microbial spoilage is due to unwanted molds [179] causing health issues and also massive economic losses for both food industry and consumers. Fermentation with LAB with selected strains or incorporation of purified microbial metabolites [180] may possess bioprotective potential against pathogenic bacteria, fungi, and mycotoxins, as reviewed in Dalié et al. [181].

In general, the scientific knowledge on the effects of fermentation on the protein digestibility and mineral solubility of quinoa and other pseudocereals is lacking; this information would further enhance the adaptation of novel crops in new geographical areas. However, utilizing the botanical biodiversity of resource-based economies in our attempts to increase the sustainability of food systems should always be carefully considered. Furthermore, there is a large gap of knowledge regarding the nutritional and health effects of fermented foods in humans, and especially the noninvasive methodology aimed to enlighten the in vivo bioavailability of plant proteins urgently needs further validation and harmonization.

## 7. Conclusions

Based on the literature surveyed for this review, food fermentations provide effective means to improve the nutritional quality of legume- and cereal-based foods, regarding dietary protein and micronutrients. Therefore, with the developments of food fermentations, the goals of sustainable development can be supported by enhancing the biodiversity of protein sources and the accessibility of healthy and nutritionally well-balanced stable foods suitable for many types of consumer groups worldwide.

Indeed, in respect to both mineral solubility and protein digestibility in legume- and cereal-based products, evidence supporting the observation that microbial fermentation has the potential to reduce phytic acid and protease inhibitory activities is accumulating. Furthermore, combination of germination and fermentation and/or addition of external phytase enzymes can provide effective means to decrease the amount of phytic acid and to increase mineral solubility in plant-derived raw materials. Combining different fermented plant proteins, instead, may help to fill the gaps, for example, in the end-product’s amino acid profile. In the future, genetic modifications of fermenting microbiota could aid the further improvements of nutritional quality parameters of fermented plant-based products; however, these types of modifications might be questionable regarding the principles of sustainable development and should be considered carefully for the sake of product quality and consumer safety.

## Supplementary Materials

The following are available online at https://www.mdpi.com/2072-6643/12/4/1020/s1, Table S1: The nutritional values of grain legumes chickpea, common bean, faba bean, narrowleaf lupin, pea, and soybean according to Fineli the National Food Composition Database of the Finnish institute for health and welfare. Table S2: The nutritional values of cereal grains oats, rye, wheat, and sorghum, and pseudocereal quinoa, according to Fineli the National Food Composition Database of the Finnish institute for health and welfare.
