# Supplementary material for: Harnessing Microbes for Sustainable Development: Food Fermentation as a Tool for Improving the Nutritional Quality of Alternative Protein Sources
Source: Nutrients. 2020 Apr 8;12(4):1020. doi: 10.3390/nu12041020 (PMC7230334; doi:10.3390/nu12041020)
Supplement: Supplementary file 1 [file nutrients-12-01020-s001.pdf]

Table S1. The nutritional values of grain legumes chickpea, common bean, faba bean, narrowleaf lupin, pea, and soybean according to Fineli the National Food Composition Database of the Finnish institute for health and welfare [1-6]. The values are expressed on a dry matter basis. n.a., not available

	Energy, calculated	Protein (g/100g)	Fat (g/100g)	Carbohydrate, available (g/100g)	Fiber, total (g/100 g)
**chickpea**	343 kcal (1436 kJ)	21.3	5.4	46.4	10.7
**common bean, black**	318 kcal (1330 kJ)	21.2	0.9	47.8	15.5
**common bean, brown or white**	305 kcal (1287 kJ)	22.2	1.5	39.5	21.6
**faba bean**	331 kcal (1384 kJ)	28.5	1.9	42.2	13.6
**narrowleaf lupin**	291 kcal (1220 kJ)[a]	33.8[b]	13.6[b]	31.0[a]	39.9[b]
**pea**	245 kcal (1025 kJ)	19.4	1.6	27.2	20.0
**soybean**	374 kcal (1565 kJ)	35.9	17.7	10.3	15.7
	**Ca (mg/100g)**	**Fe (mg/100g)**	**K (mg/100g)**	**Mg (mg/100g)**	**Zn (mg/100g)**
**chickpea**	160.0	5.5	1 000.0	130.0	3.0
**common bean, black**	160.0	8.7	1 500.0	160.0	2.2
**common bean, brown or white**	139.5	5.2	1 285.0	157.5	0.0
**faba bean**	103.0	6.7	1 062.0	192.0	3.1
**narrowleaf lupin**	152.4[c]	4.2[c]	1294.4[c]	205.5[c]	3.7[c]
**pea**	64.0	4.8	918.0	110.0	2.9
**soybean**	163.0	8.4	1 730.0	225.0	1.0
	**Folate, total (µg/100g)**	**B6 (mg/100 g)**	**Niacin (mg/100)**	**Riboflavin (mg/100g)**	**Thiamin (mg/100g)**
**chickpea**	180.0	0.53	5.2	0.24	0.39
**common bean, black**	444.0	0.29	6.2	0.19	0.90
**common bean, brown or white**	441.0	0.47	6.0	0.20	0.55
**faba bean**	78.8	0.44	7.0	0.35	0.55
**narrowleaf lupin**	n.a.	n.a.	n.a.	0.24[d]	0.71[d]
**pea**	33.0	0.16	6.2	0.07	0.62
**soybean**	370.0	0.46	9.7	0.31	1.10

[a] Nalle et al. 2011 [7]; [b] Lqari et al. 2002 [8]; [c] Porres et al. 2007 [9]; [d] Torres et al. 2005 [10]

Table S2. The nutritional values of cereal grains oats, rye, wheat, and sorghum, and pseudocereal quinoa, according to Fineli the National Food Composition Database of the Finnish institute for health and welfare [11-15]. The values are expressed on a dry matter basis.

	Energy, calculated	Protein (g/100g)	Fat (g/100g)	Carbohydrate, available (g/100g)	Fiber, total (g/100g)
**oat flour**	390 (1634 kJ)	12.6	7.1	63.9	9.0
**rye flour, wholemeal**	324 (1355 kJ)	9.6	1.9	59.4	13.9
**wheat flour, *T. aestivum***	347 (1454 kJ)	11.9	1.3	69.1	3.5
**wheat flour, durum, wholemeal**	358 (1498 kJ)	13.0	2.5	63.8	12.6
**sorghum, grain**[a]	339 (1419 kJ)	11.3	3.3	68.3	6.3
**quinoa, seed**	347 (1454 kJ)	11.9	6.1	57.2	7.0
	**Ca (mg/100g)**	**Fe (mg/100g)**	**K (mg/100g)**	**Mg (mg/100g)**	**Zn (mg/100g)**
**oat flour**	56.0	5.5	457.0	160.0	4.1
**rye flour**	33.0	3.3	445.0	97.0	2.9
**wheat flour, *T. aestivum***	22.0	0.8	189.0	40.0	1.1
**wheat flour, durum, wholemeal**	34.0	3.5	431.0	144.0	4.2
**sorghum, grain**	28.0[a]	4.4[a]	350[a]	228.5[b]	2.7[c]
**quinoa, seed**	47.0	4.6	563.0	197.0	3.1
	**Folate, total (µg/100g)**	**B6 (mg/100 g)**	**Niacin (mg/100)**	**Riboflavin (mg/100g)**	**Thiamin (mg/100g)**
**oat flour**	38.7	0.13	4.3	0.09	0.33
**rye flour**	38.0	0.17	2.0	0.16	0.31
**wheat flour, *T. aestivum***	17.0	0.10	3.1	0.05	0.08
**wheat flour, durum, wholemeal**	43.0	0.42	9.4	0.12	0.42
**sorghum, grain**	20[d]	0.21[d]	2.9[a]	0.1[a]	0.2[a]
**quinoa, seed**	92.0	0.49	4.3	0.32	0.36

[a] SELF Nutrition Data [16]; [b] Makokha et al. 2002 [17]; [c] Kumar et al. 2010 [18]; [d] Ochanda et al. 2010 [19], calculated as mean for red and white sorghum

References

1. Fineli the National Food Composition Database of the Finland. Bean, black bean, turtle bean, dried *Phaseolus vulgaris*. https://fineli.fi/fineli/en/elintarvikkeet/35635?q=papu%2520kuivattu&foodType=ANY&portionUnit=G&portionSize=100&sortByColumn=points&sortOrder=asc&component=2331&, Accessed: February 27 2020.
2. Fineli the National Food Composition Database of the Finland. Bean, common bean, brown bean, white bean, average *Phaseolus vulgaris*. https://fineli.fi/fineli/en/elintarvikkeet/373, Accessed: February 27 2020.
3. Fineli, the National Food Composition Database in Finland. Chick pea, dried *Cicer arietinum*. https://fineli.fi/fineli/en/elintarvikkeet/31218, Accessed: February 27 2020.
4. Fineli, the National Food Composition Database in Finland. Soya beans, dried *Glysine max*. https://fineli.fi/fineli/en/elintarvikkeet/391?q, Accessed: February 27 2020
5. Fineli, the National Food Composition Database of Finland. Bean, broad bean, fava bean, dry, broad bean groats *Vicia faba*. https://fineli.fi/fineli/en/elintarvikkeet/34743?q=papu%2520kuivattu&foodType=ANY&portionUnit=G&portionSize=100&sortByColumn=points&sortOrder=asc&component=2331&, Accessed: February 27 2020.
6. Fineli, the National Food Composition Database of Finland. Pea, green, dried *Pisum sativum*. https://fineli.fi/fineli/en/elintarvikkeet/371?q, Accessed: February 27 2020.
7. Nalle, C.L.; Ravindran, V.; Ravindran, G. Nutritional Value of Narrow-Leafed Lupin (*Lupinus Angustifolius*) for Broilers. Br. Poult. Sci. **2011**, *52*, 775-781.
8. Lqari, H.; Vioque, J.; Pedroche, J.; Millán, F. *Lupinus Angustifolius* Protein Isolates: Chemical Composition, Functional Properties and Protein Characterization. Food Chem. **2002**, *76*, 349-356.
9. Porres, J.M.; Aranda, P.; López-Jurado, M.; Urbano, G. Nitrogen Fractions and Mineral Content in Different Lupin Species (*Lupinus Albus*, *Lupinus Angustifolius*, and *Lupinus Luteus*). Changes Induced by the α -Galactoside Extraction Process. J. Agric. Food Chem. **2007**, *55*, 7445-7452.
10. Torres, A.; Frias, J.; Vidal-Valverde, C. Changes in Chemical Composition of Lupin Seeds (*Lupinus Angustifolius*) After Selective α-galactoside Extraction. J. Sci. Food Agric. **2005**, *85*, 2468-2474.
11. Fineli the National Food Composition Database of the Finland. Flour, oat flour *Avena sativa*. https://fineli.fi/fineli/en/elintarvikkeet/30190?q=oat%20flour&foodType=ANY&portionUnit=G&portionSize=100&sortByColumn=points&sortOrder=asc&component=2331&, Accessed: February 27 2020.
12. Fineli the National Food Composition Database of the Finland. Flour, rye, wholegrain rye flour *Secale cereale*. https://fineli.fi/fineli/en/elintarvikkeet/100?q=rye%20flour&foodType=ANY&portionUnit=G&portionSize=100&sortByColumn=points&sortOrder=asc&component=2331&, Accessed: February 27 2020.
13. Fineli the National Food Composition Database of the Finland. Flour, wheat flour, semi-coarse, V600 *Triticum aestivum*. https://fineli.fi/fineli/en/elintarvikkeet/34242?q=triticum%2520aestivum&foodType=ANY&portionUnit=G&portionSize=100&sortByColumn=points&sortOrder=asc&component=2331&, Accessed: February 27 2020.
14. Fineli the National Food Composition Database of the Finland. Flour, whole wheat flour *Triticum durum* desf. https://fineli.fi/fineli/en/elintarvikkeet/28905?q=flour%20durum&foodType=ANY&portionUnit=G&portionSize=100&sortByColumn=points&sortOrder=asc&component=2331&, Accessed: February 27 2020.
15. Fineli the National Food Composition Database of the Finland. Quinoa *Chenopodium quinoa* willd. https://fineli.fi/fineli/en/elintarvikkeet/31175, Accessed: February 27 2020.
16. SELF Nutrition Data. Sorghum nutrition facts & calories. https://nutritiondata.self.com/facts/cereal-grains-and-pasta/5732/2, Accessed: February 27 2020.
17. Makokha, A.O.; Oniang'o, R.K.; Njoroge, S.M.; Kamar, O.K. Effect of Traditional Fermentation and Malting on Phytic Acid and Mineral Availability from Sorghum (*Sorghum Bicolor*) and Finger Millet (*Eleusine Coracana*) Grain Varieties Grown in Kenya. Food and nutrition bulletin **2002**, *23*, 241-245.
18. Kumar, A.A.; Reddy, B.V.; Sahrawat, K.L.; Ramaiah, B. Combating Micronutrient Malnutrition: Identification of Commercial Sorghum Cultivars with High Grain Iron and Zinc. J. SAT Agric. Res. **2010**, *8*.
19. Ochanda, S.O.; Akoth, O.C.; Mwasaru, A.M.; Kagwiria, O.J.; Mathooko, F.M. Effects of Malting and Fermentation Treatments on Group B-Vitamins of Red Sorghum, White Sorghum and Pearl Millets in Kenya. J. Appl. Biosci. **2010**, *34*, 2128-2134.
